# Sociodemographic Characteristics and Inequities Associated With Access to In-Person and Remote Elementary Schooling During the COVID-19 Pandemic in New York State

**DOI:** 10.1001/jamanetworkopen.2021.17267

**Published:** 2021-07-15

**Authors:** Ashley M. Fox, Jun Soo Lee, Lucy C. Sorensen, Erika G. Martin

**Affiliations:** 1 Department of Public Administration and Policy, University at Albany, Albany, New York; 2 Department of Economics, University at Albany, Albany, New York; 3 Center for Collaborative HIV Research in Practice and Policy, University at Albany, Albany, New York

## Abstract

This cross-sectional study compares in-person school reopening decisions by student sociodemographic characteristics among elementary schools in New York State.

## Introduction

School districts confronted difficult reopening decisions in Fall of 2020 after pivoting to remote learning in March 2020 because of the COVID-19 pandemic.^1^ In its kindergarten to 12th grade school reopening guidance, the National Academies highlighted how uneven reopening plans might exacerbate existing economic and social inequalities among students and recommended prioritizing full-time, in-person reopening for students in kindergarten through fifth grade and students with special needs.^2^ New federal guidance about safe, in-person instruction cautions that remote-only education may disproportionately disadvantage low-income children.^3^ We compare in-person school reopening decisions in October 2020 by student sociodemographic characteristics in New York State (NYS), which was an early epicenter of the COVID-19 pandemic. We focus on elementary schools because learning loss from remote education may be especially acute among this age group^2,3^ and COVID-19 transmission is lower among younger children.^1^

## Methods

This cross-sectional study was deemed exempt from institutional review board approval or informed consent because the collected data are based on publicly available information, and reidentification was not possible. This study followed the Strengthening the Reporting of Observational Studies in Epidemiology (STROBE) reporting guideline.

To document potential inequalities in school reopening plans, we used an ecological cross-sectional design to assess variation in NYS students’ access to in-person schooling in their district by sociodemographic characteristics. Our sample comprised all 1 140 540 NYS students in 2498 public elementary schools clustered within 704 school districts. The outcome measure of being in a school district with an in-person reopening plan as of October 12, 2020, was accessed from the COVID-19 School Response Dashboard.^4^ Aggregate sociodemographic characteristics were obtained from administrative databases, including district-level urbanicity, student racial and ethnic composition, and percentage of students who were from a family with low income, were English-language learners, students with homelessness, and students with disabilities. We adjusted our estimates of access to in-person schooling by the rate of COVID-19 deaths per 100 000 population in August 2020 to assess the COVID-19 burden as an explanatory factor for school districts’ reopening decisions.

An examination of race and ethnicity was included in this study because assessing disparities in access to in-person schooling was a major objective. Secondary data on student race and ethnicity were accessed from the 2018 to 2019 New York State Education Department (NYSED) Enrollment Database. All schools in the U.S. are required to report student race and ethnicity counts according to federal reporting guidelines for submission to National Center for Education Statistics. The NYSED Enrollment Database collects information from school districts on school characteristics including students’ race and ethnicity. Information is based on the race(s) and ethnicity to which the student primarily identifies and is indicated by the student or their parent or guardian. If the student, parent or guardian, or staff member does not designate race and ethnicity, a school administrator makes the best determination for reporting purposes. The categories we used in our analysis aligned with the standard reporting categories required of all schools across the country and included Black or African American (not of Hispanic origin), Hispanic or Latino, White (not of Hispanic origin), and all other races. Although there are important differences among students who were included in the other category (ie, Asian, American Indian or Alaskan Native, and 2 or more races groups) that are masked when these groups are combined, only 3.7%, 0.6%, and 3.5% of NYS elementary students were in these groups, respectively. We did not reclassify students reporting 2 or more races to our category of Black and/or Hispanic because we did not have information on specific races to which they identified.

Statistical analyses were 2-tailed and performed using Stata 16.1/14.2 (StataCorp). Secondary data for school reopening plans and district characteristics as of October 2020 were assembled between December 2020 to March 2021. Statistical significance was set at *P* < .05. The eAppendix in the Supplement contains additional Methods details.

## Results

**Figure 1.**
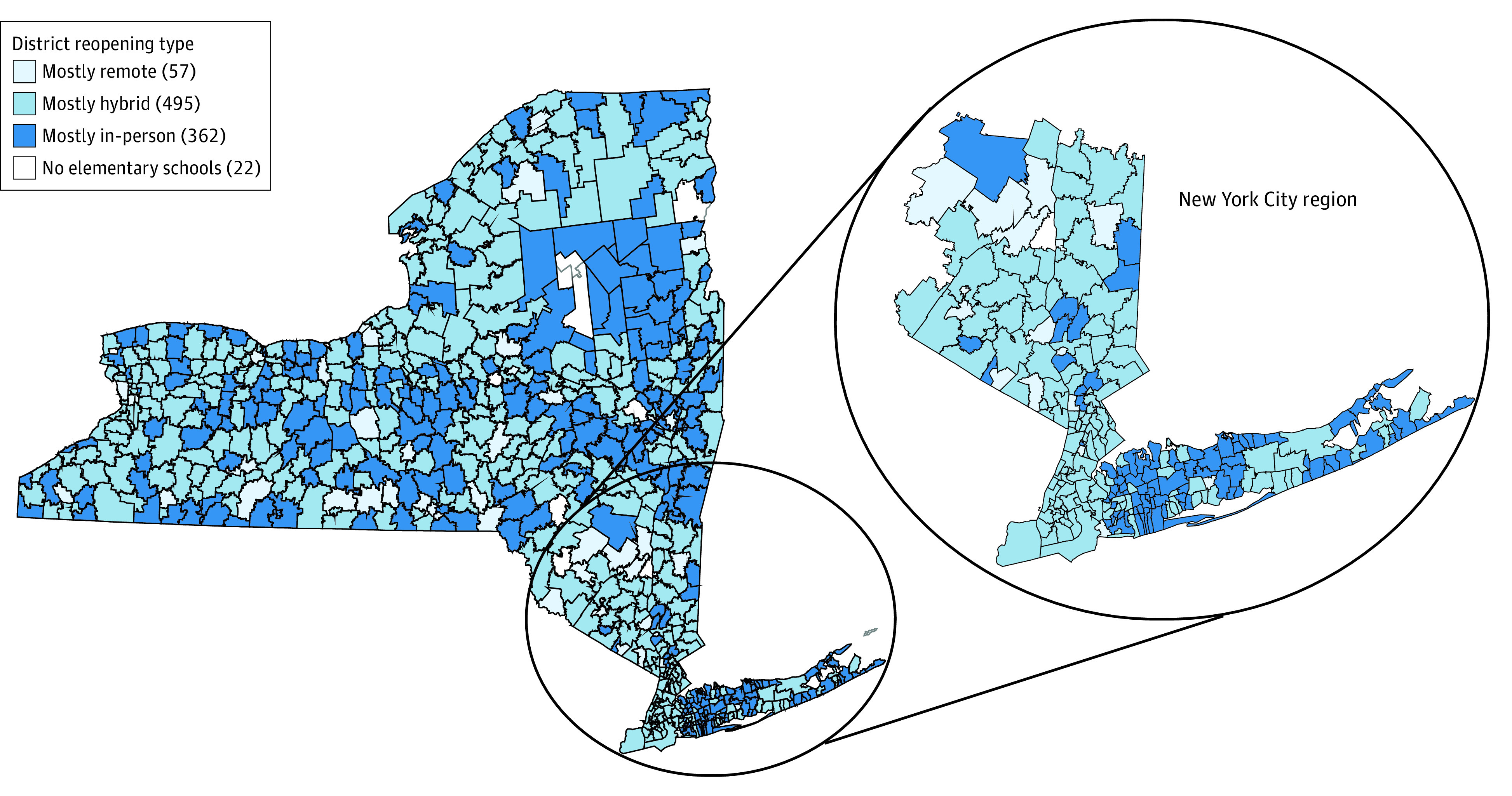
New York State (NYS) School District Reopening Plans as of October 12, 2020

Of the 1 140 540 students included in this study, 554 013 (48.6%) were female students, 492 167 (43.2%) were White students, and 161 329 (14.1%) were Black students. The cohort consisted of 208 416 students (18.3%) with a disability, 49 488 (4.3%) with homelessness, 658 205 (57.7%) with low income, and 131 002 (11.5%) students who were English language learners. By mid-October 2020, 271 of 704 districts (38.5%) had elementary schools that were open full-time with in-person learning. Figure 1 maps school districts by elementary school reopening types. While 343 303 (30.1%) White students had access to in-person schooling, only 92 384 (8.1%) Hispanic students and 60 449 (5.3%) Black students had access to in-person schooling. Students with low-income, were English language learners, with homelessness, with a disability, or living in urban areas were each less likely to have access to in-person instruction compared with their counterparts who were not disadvantaged (low income: 119 757 [10.5%] vs 326 194 [28.6%]; English language learner: 74 135 [6.5%] vs 224 686 [19.7%]; with homelessness: 31 935 [2.8%] vs 215 562 [18.9]; with disabilities: 164 238 [14.4%] vs 216 703 [19.0%]; urban vs rural/suburban: 44 481 [3.9%] vs 809 783 [35.5%]).

**Figure 2.**
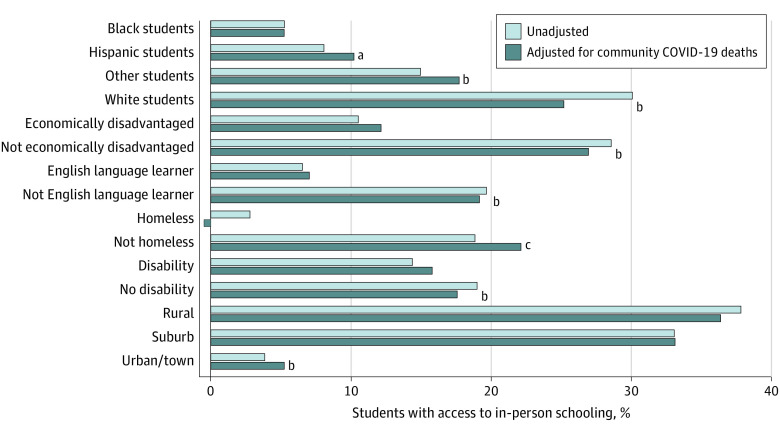
Likelihood of Full-Time In-Person School Access by Student Characteristics, Adjusted and Unadjusted for COVID-19 Deaths The unadjusted bars represent the proportion of elementary students in each sociodemographic group with access to in-person schooling in Fall 2020. The adjusted bars are based on models that control for the COVID-19 mortality rate at the county level in August 2020, as a measure of community disease burden that may have influenced schools' reopening plan decisions. The adjusted percentage of students with homelessness who had access to in-person schooling is −0.5%; this is an artifact of the adjustment methods. The asterisks represent the significance levels of χ^2^ tests of the differences in adjusted proportions between groups of students. After adjusting for COVID-19 rates, differences that were still significant are indicated with asterisks based on the following reference groups: for race/ethnicity categories, Black students are the reference group; for sociodemographic characteristics, students having that characteristic are the reference group (ie, being economically disadvantaged, English language learners, having homelessness, and students with a disability); and for geographic categories, students in rural districts are the comparison group. Racial and ethnic groups categorized as other included American Indian or Alaska Native (not of Hispanic origin), Asian, Native Hawaiian, or other Pacific Islander (not of Hispanic origin), and 2 or more races (not of Hispanic origin). ^a^*P* < .10. ^b^*P* < .01. ^c^*P* < .05.

The proportion of students in districts with elementary schools that were open full-time with in-person learning by student characteristics is shown in Figure 2. The adjusted bars show the proportion of students who would have had access to in-person schooling if all communities experienced the same level of COVID-19 burden. While there were numerical differences, the patterns of differences between groups were similar, suggesting that local COVID-19 burden was not a factor that influenced plans. Although the proportion of White students with access to in-person schooling reduced from 30.1% to 25.2% after adjusting for community COVID-19 deaths, the unadjusted vs adjusted proportion of Black students with access to in-person schooling remained nearly constant at 5.2% vs 5.3% (Figure 2).

## Discussion

Among the few school districts providing full-time in-person elementary school instruction, most districts served predominately White students, rural/suburban students, and children who were not disadvantaged (ie, children who were not from a low-income family, were not English language learners, did not have homelessness, and did not have a disability). Local variation in COVID-19 burden was not found to be an explanatory factor for reopening decisions. These findings support concerns that differences in school reopening plans may exacerbate existing achievement gaps for students from disadvantaged backgrounds. It also confirms that different reopening plans were associated with the districts’ capacity to reopen safely rather than level of community infection, both of which are closely tied to socioeconomic factors.^2,5^ Evidence from March 2021^5^ suggests that students lost nearly one-third of a year’s learning between Fall 2019 and Fall 2020 and that the test score declines of Black students were approximately 50% larger than White students, with the highest achievement declines in districts with fully remote instruction. Beyond implications for learning, in-person instruction is vital to provide vulnerable students with access to technology, nutrition, physical health, and behavioral health services.^6^ Study limitations include a cross-sectional ecological design that may mask variation within districts, generalizability beyond NYS, and reliance on administrative data, which may have misclassifications.

## Notes

Supplement. **eAppendix.** Supplementary Methods
