# Supplementary material for: Sociodemographic Characteristics and Inequities Associated With Access to In-Person and Remote Elementary Schooling During the COVID-19 Pandemic in New York State
Source: JAMA Netw Open. 2021 Jul 15;4(7):e2117267. doi: 10.1001/jamanetworkopen.2021.17267 (PMC8283559; doi:10.1001/jamanetworkopen.2021.17267)
Supplement: Supplement. — eAppendix. Supplementary Methods [file jamanetwopen-e2117267-s001.pdf]

Supplemental Online Content

Fox AM, Lee JS, Sorensen LC, Martin EG. Sociodemographic characteristics and inequities associated with access to in-person and remote elementary schooling during the COVID-19 pandemic in New York state. *JAMA Netw Open*. 2021;4(7):e2117267 doi:10.1001/jamanetworkopen.2021.17267

eAppendix. Supplementary Methods

This supplemental material has been provided by the authors to give readers additional information about their work.

© 2021 Fox AM et al. *JAMA Network Open.*

eAppendix. Supplementary Methods

This document provides further detail on our underlying data and methods for calculating the unadjusted and adjusted proportions of students with access to in-person schooling for each defined student population.

Data and Measures

School reopening data (outcome measure). The outcome measure was being in a school district with a re-opening plan for primarily in-person instruction as of October 12, 2020. These data came from the COVID-19 School Report Card developed by the New York State Department of Health for every public school in the state. Schools are responsible for submitting this information to the state health department, which compiles and makes available these data for public access.

We downloaded data on school re-opening plans from the COVID-19 School Response Dashboard website ("wave 4" data, corresponding to the time period of October 12-25, 2020).[1] The school reopening plan date of October 12th, 2020 was selected because it was the earliest available data that incorporated school re-opening plans for New York City Schools. New York City Schools re-opened according to their reopening plans in October 2020. School reopening plans were due to the New York State Department of Education by July 31, 2020 so that the State could make decisions in early August 2020.[2] Although these data are from a few months later, we believe these are nonetheless a good reflection of early plans in the Fall of 2020 as school districts were making adjustments early in the school year as the pandemic and scientific knowledge about in-person schooling evolved, and as school district administrators gained experience with providing in-person versus remote instruction.

We restricted the sample to public schools that were either elementary-only or else containing elementary school grades. Our analysis took an ecological approach at the school district level. To develop our measure of "access to in-person schooling," we aggregated to the school district level rather than the school level because school re-opening plans were decided at a district level, not a school level. In the overwhelming majority of cases, the re-opening decisions (i.e., fully in-person, hybrid, fully remote) apply to all elementary schools in a given district. We excluded charter schools because they are typically not contained within a certain school district, and because charter schools developed their own reopening plans separately from the district reopening plans.

[1] https://covidschooldashboard.com, accessed on December 21, 2020.
[2] https://tinyurl.com/5fpnnjyt

© 2021 Fox AM et al. *JAMA Network Open.*

The COVID-19 School Response Dashboard data identifies three types of reopening plans among schools: 1) full-time in-person, 2) hybrid, and 3) fully remote. We assigned scores to each of these reopening plans as follows: 0 if the reopening plan was remote-only, 0.5 if the reopening plan was hybrid, and 1 if the reopening plan was fully in-person. Generally, all elementary schools within a district had the same reopening plan which we verified with our hand search. Where this was not the case, we created a district-level score by taking the average of the school-level scores with an equal weight to each school. We then classified districts into three categories based on their overall scores: 1) mostly in-person (score > 0.66), 2) mostly hybrid (score between 0.33 and 0.66), and 3) mostly remote (score <0.34). The decision for this cutoff was based on our exploratory data analysis of the distribution of district-level scores and these three groups emerging as clear patterns in the data, and results were robust to the use of different cutoffs.

For the descriptive maps, we retained these three groupings. For the statistical analysis, we developed a dichotomous measure of mostly in-person versus other schooling (mostly hybrid and mostly remote).

Education data (predictors). The following measures, aggregated for all elementary schools to the school district level, comprised the student characteristics of interest as predictors of access to in-person schooling: student race and ethnicity (mutually exclusive categories of Black non-Hispanic, Hispanic, White non-Hispanic, and all other races and ethnicities combined), economic disadvantage, English Language Learner status, homeless status, disability status, and region type (urban/town, suburb, and rural). These characteristics were from the 2018-2019 New York State Education Department (NYSED) Enrollment Database, available at https://data.nysed.gov/downloads.php, and the 2018-2019 National Center for Education Statistics (NCES) Common Core of Data School and District Directory Files, available at https://nces.ed.gov/ccd/files.asp. Additional details on specific measures are below:

> *Race and ethnicity* (data source: NYSED Enrollment). All schools in the U.S. are required to report student race and ethnicity counts according to federal reporting guidelines for submission to NCES. The New York State Education Department (NYSED) Enrollment Database collects information from school districts on school characteristics including students' race and ethnicity. Information is based on the race(s) and ethnicity to which the student primarily identifies and is indicated by the student or their parent/guardian. If the student, parent/guardian, or staff member does not designate race and ethnicity, a school administrator makes the best determination for reporting purposes. The race categories provided as options are: "Black or African American (Not of Hispanic Origin)," "Hispanic or Latino," "White (Not of Hispanic Origin)," "American Indian or Alaska Native (Not of Hispanic Origin)," "Asian, Native Hawaiian, or Other Pacific Islander (Not of Hispanic Origin)," and "Two or More Races (Not of Hispanic Origin)." These standard reporting categories are required of all schools across the country. For our analysis, we used the categories of: 1) "Black or African American (Not of Hispanic Origin)," 2) "Hispanic or Latino," 3) "White (Not of Hispanic Origin)," and 4) all other races. Although there are important differences across students in the Asian, American Indian or Alaskan

© 2021 Fox AM et al. *JAMA Network Open.*

Native, and Two or More Races groups that are masked when these groups are combined, only 3.7%, 0.6%, and 3.5% of NYS elementary students are in these groups, respectively. We did not reclassify students reporting “two or more races” to our category of Black and/or Hispanic because we did not have information on specific races to which they identified.

- *Economic disadvantage* (data source: NYSED Enrollment). Economically-disadvantaged students are defined in the data as, “thosewho participate in, or whose family participates in, economic assistance programs, such as the free or reduced-price lunch programs, Social Security Insurance (SSI), food stamps, foster care, refugee assistance, Earned Income Tax Credit (EITC), Home Energy Assistance Program (HEAP), Safety Net Assistance (SNA), Bureau of the Indian Affairs (BIA), or Temporary Assistance for Needy Families (TANF)” (NYSED, 2021). The school district is responsible for validating and reporting data on student economic disadvantage.
- *English Language Learner status* (data source: NYSED Enrollment). These data are based on a code that the student has received English Language Learner (ELL) services in NYS public schools, as evidenced by having been reported with Program Service codes 5709 (English as a New Language), 5676 (Transitional Bilingual Education Program) or 5687 (One Way or Two Way Dual Language Program).
- *Homeless status* (data source: NYSED Enrollment). This is based on a code that indicates the primary nighttime residence of students who are identified as homeless by educators, students, or families. This administrative information is typically collected and reported by the Local Education Agency (LEA)'s homeless liaison, according to regulations under the McKinney-Vento Homeless Assistance Act.
- *Disability status* (data source: NYSED Enrollment). This is defined as the number of school-age children receiving special education services as of October of the 2018-2019 school year. It includes students with the following forms of disability: autism, emotional disturbance, learning disability, intellectual disability, deafness, hearing impairment, speech or language impairment, visual impairment (includes blindness), orthopedic impairment, other health impairment, multiple disabilities, deaf-blindness, and traumatic brain injury.
- *Urbanicity* (data source: NCES Common Core of Data). These data reflect whether a school is located in one of 12 urban-centric locale types: city, large; city, midsize; city, small; suburb, large; suburb, midsize; suburb, small; town, fringe; town, distant; town, remote; rural, fringe; rural, distant; and rural, remote. These were collapsed into three categories of urban/town, suburban, and rural.

COVID-19 mortality data (covariate). We calculated daily county level COVID-19 deaths per 100,000 population. Daily August 2020 COVID-19 mortality data are from the *New York Times*[3], which at the time of analysis was widely considered as one of the most comprehensive and

[3] https://github.com/nytimes/covid-19-data, accessed on January 15, 2021.

reliable sources of aggregated mortality data. County population is based on 2019 annual estimates of the resident population for counties from Census[4].

Sources of bias. Like all administrative data, there is a possibility of misclassification which might lead to bias. We did not adjust for potential bias in reporting because we did not have sufficient information to know the potential direction of bias and how it might influence conclusions.

Missing data and recoding of school types. We have no missing data within this sample; i.e., all districts in the sample had information on reopening plans, urbanicity, and student demographics.[5] However, some non-standard school districts were excluded from the study sample.

There are 1,068 districts in NYS according to the NCES Common Core of Data district directory file. After exclusion of non-standard district types, including 298 independent charter districts, 37 service agencies, 6 state agencies, and 1 supervisory union administrative center, there are a total of 726 regular public school districts.[6] When merging the school reopening data from COVID-19 School Response Dashboard, there were a few discrepancies between the school types listed in the Dashboard and their characteristics in the NYSED Enrollment Data. Three districts were defined as having elementary schools in the COVID-19 School Response Dashboard but not in the NYSED Enrollment Data; we retained their classification as having elementary schools.

In addition, 77 school districts were recorded in the COVID-19 School Response Dashboard as having no elementary schools, but instead as having school levels labeled as "other" grade configurations. These districts tended to have combined schools, such as K-8 grade levels, instead of K-5, and therefore we included any schools other than the exclusively middle or high schools from 55 of these 77 districts. The remaining 22 districts contained no elementary schools, and thus were excluded entirely from the analysis. In the map, these schools are represented as not having elementary schools. Our final analytical sample contains a total of 704 school districts across NYS.

Analytic Methods

Access to in-person schooling. Although our aggregated predictor and outcome data are at the school and district levels, we are interested in access to in-person schooling from the perspective of the individual student (i.e., the likelihood of access to in-person instruction in elementary schools within students' districts, comparing students with different

---

[4] https://www.census.gov/data/tables/time-series/demo/popest/2010s-counties-total.html}{https://www.census.gov/data/tables/time-series/demo/popest/2010s-counties-total.html

[5] There were missing county FIPS code for 37 school districts, which were required to merge the school district information on reopening plans with the COVID-19 mortality data. These were inputted by manually searching up the addresses and zip codes for schools within those districts.

[6] The supervisory union administrative center is the NYC Chancellor's Office, and the six state agencies are the NYS Office for People with Developmental Disabilities, the NYS School for the Deaf, the NYS Department of Corrections, the NYS Office of Mental Health, the NYS School for the Blind, and the NYS Office of Children and Family Services.

© 2021 Fox AM et al. *JAMA Network Open.*

characteristics). To provide this interpretation of the data, we use the district-level data to calculate the proportion of students with different characteristics having access to in-person schooling (outcome measure). We calculate both an unadjusted proportion, and a proportion adjusted for community differences in COVID-19 mortality that may be associated with reopening plans.

Unadjusted proportions method. We first calculate the raw proportion (P) of elementary school students having some characteristic k with access to in-person schooling in their district d as follows:

$$P_k = \frac{\sum_d n_{dk} \times \mathbb{I}(R_d = 1)}{\sum_d n_{dk}}$$

In this equation, n_{dk} equals the number of elementary school students in district d with characteristic k, and R_d represents district re-opening type defined as follows:

$$R_d = \begin{cases} 1 \; if \; most \; elementary \; schools \; have \; in-person \; schooling \\ 0 \; if \; most \; elementary \; schools \; have \; remote \; or \; hybrid \; schooling \end{cases}$$

Adjusted proportions method. A potential explanation for variation in reopening plans is the local COVID-19 mortality, as communities with higher disease burden may have been less likely to have in-person schooling. While there are multiple measures of disease burden, we decided to use mortality because there was considerable under-reporting of COVID-19 testing in the earlier period of the pandemic and mortality is less sensitive to issues with insufficient testing capacity. Our adjusted proportions residualize all variables by the local COVID-19 mortality.

To calculate rates adjusted for the community COVID-19 mortality rate, we use a bootstrapping approach with 500 resamplings, clustered at the school district level. We cluster the bootstrap at the school district level because decisions regarding school reopening plans were typically made by the school district. Within each of these bootstrap resamplings, separately for each student characteristic *k* where *k* specifies a student race/ethnicity category, school urbanicity category, or other student socioeconomic characteristic as described above, we conduct the following four steps:

1. Simulate a student-level dataset based on district access to in-person schooling and the proportion of elementary school students in district *d* that possess characteristic *k*. For example, in a district with 100 total elementary school students and a 40 percent economic disadvantage rate, we would construct 40 economically-disadvantaged student observations and 60 non-economically-disadvantaged student observations. In a rural district with 300 elementary school students, we would construct 300 student observations and label them all as attending school in a rural area.

2. With this student-level dataset, we residualize the in-person schooling indicator by the community COVID mortality rate:

© 2021 Fox AM et al. *JAMA Network Open.*

$$R_{id} = \alpha_1 M_d + \varepsilon_{id}$$
$$\widehat{R_{id}^r} = R_{id} - \widehat{\alpha_1} M_d$$

In the equations above, R_{id} is an indicator of whether or not simulated student *i* has access to in-person schooling in district *d*, M_d is a county measure of the rate of COVID-19 deaths per 100,000 population per day, and $\widehat{R_d^r}$ is the residualized in-person schooling indicator. The goal of this step is to remove the variation in in-person school reopening that is correlated with community COVID-19 deaths.

3. We then also residualize the indicator of whether or not the simulated student possesses characteristic *k* by the community COVID mortality rate:

$$Y_{idk} = \alpha_2 M_d + \varepsilon_d$$
$$\widehat{Y_{idk}^r} = Y_{idk} - \widehat{\alpha_2} M_d$$

In this equation, county mortality is defined as above, Y_{idk} is an indicator of whether student *i* in district *d* possesses characteristic *k*, and $\widehat{Y_{idk}^r}$ is the estimated residual. Again, the goal of this step is to remove the variation in student and community characteristics that are associated with community COVID-19 deaths.

4. Finally, we calculate the statewide proportion of students with characteristic *k* with access to in-person schooling in their elementary schools, adjusted for community COVID-19 mortality:

$$\widehat{P_k} = \frac{\sum_i \widehat{R_{id}^r} \times \widehat{Y_{idk}^r}}{\sum_i \widehat{Y_{idk}^r}}$$

In this fraction, the numerator equals the sum across the entire state of the residualized in-person schooling indicator multiplied by the student characteristic *k* indicator. In words, the numerator equals the total number of elementary students of characteristic *k* with access to in-person schooling in the state, adjusted for statewide patterns of COVID-19 mortality. The denominator equals the sum across the entire state of the residualized student characteristic *k* indicator, or in words, the total number of students that have that characteristic, again adjusted for patterns of COVID-19 mortality.

In essence, this approach allows us to capture the distribution of student types across districts that is unrelated to patterns of COVID community spread. Following this process, we calculated each adjusted proportion as a simple average of the adjusted proportions from the 500 bootstraps. The final step is to recalibrate these rates such that the average proportion of students with in-person schooling remains constant with the unadjusted proportions.

© 2021 Fox AM et al. *JAMA Network Open.*

Chi-square tests to compare outcomes of access to in-person schooling by student characteristic. The bootstrapping procedure outlined above allows us to assess the degree of statistical uncertainty in the adjusted proportions of access to in-person schooling by student characteristic. In particular, following the bootstrapping procedure, we can perform Chi-square tests for the null hypothesis that the true adjusted proportion of students in group *g* with access to in-person schooling equals the true adjusted proportion of students in group *h* with access to in-person schooling, conducted for all relevant comparison groups *g* and *h*. Because the bootstrap resampling was clustered at the school district level rather than at the student or school level, these Chi-square tests provide a conservative test for differences across groups.

© 2021 Fox AM et al. *JAMA Network Open.*
